# Traumatic Hyphaema: A report of 472 consecutive cases

**DOI:** 10.1186/1471-2415-8-24

**Published:** 2008-11-26

**Authors:** Adeyinka O Ashaye

**Affiliations:** 1 Department of Ophthalmology, University College Hospital, Ibadan, Nigeria

## Abstract

**Background:**

Strategies for prevention of eye injuries require knowledge of the cause of the injuries. This study was done to determine the patient characteristics, the cause of injury, and where cases of traumatic hyphaema that necessitated admission to a tertiary hospital occurred. This may enable an appropriate intervention in the prevention of such injuries.

**Methods:**

Retrospective case analysis of 472 patients with traumatic hyphaema admitted to the University College Hospital, Ibadan between January 1997 and December 2006.

**Results:**

The home was the single most frequent place of injury for all cases and for 75% of cases in children aged 0–10 years. Injuries that occurred at school comprised about one-fifth of cases. Sport-related injuries were uncommon.

The most common activities preceeding injuries were play, corporal punishment and assault. Stones, sticks and whiplash were the agents that caused traumatic hyphaema. Occupational-related hyphaema that caused injuries was mostly in farmers and artisans, few of whom used protective goggles. The majority of patients were males. Children and young adults aged ≤ 20 years comprised 63.6% of patients. A total of 336 (76%) eyes had at least one surgical intervention. While 298 (73.2%) patients had visual acuity (VA) less than 6/60 at presentation, 143 (37.0%) of eyes had visual acuity (VA) < 6/60 3 months after injury.

**Conclusion:**

The injuries leading to traumatic hyphaema occur mostly at home and school, and frequently affect children and young adolescents. Over one-third resulted in blindness in the affected eye. The focus should be on prevention of stick-related eye injuries at these locations and improving access to eye health services for patients who sustained eye injuries.

## Background

The eyes are the third most common organs affected by injuries, next to the hands and feet [1], despite the fact that they represent only 0.27% of the total body area and 4% of the facial area. Eye injuries still remain one of the most common causes of unilateral blindness worldwide. Blunt eye injuries mostly result in traumatic hyphaema and are not an infrequent cause of presentation to the emergency units of many eye clinics [2-8]. Most result from unnecessary eye injuries, which are largely preventable.

Although eye injuries are a major public health problem globally, most studies have come from developed countries. Reports from developed countries suggest that severe eye injuries take place during work and leisure [3,5,7], but reports from developing countries are different[8,9]. Besides, changing of lifestyles globally might have important effects on the patterns of blunt eye injuries.

This study was undertaken to provide information on the clinical and demographic data of patients admitted with traumatic hyphaema to the eye unit of University College Hospital (UCH), Ibadan, during a 10-year period, and to identify the cause and place of such injury. This information may help in the development of appropriate preventive measures.

## Methods

This was a retrospective study of all patients with traumatic hyphaema due to closed globe injury, who were admitted to the eye ward of UCH, Ibadan, between January 1997 and December 2006. Ibadan is the capital city of Oyo State located in the South Western region of Nigeria (geographic coordinates 7°N, 23°E). The current population is estimated at 5.5 million. Oyo State covers a land area of 27.7 km^2 ^and has an estimated population of 25 million [10]. Ibadan is an ancient city, the home of the indigenous Yoruba people. Agriculture is the predominant occupation.

The eye department of UCH serves as a major referral center for emergency and specialised eye care in Ibadan and its surrounding smaller towns and villages. In Ibadan there is another public hospital and five private eye clinics that provide both emergency and regular eye care, but the majority of patients are seen at the UCH. Access to UCH is by referral but emergency cases are seen without referral.

Patients' data were abstracted from their case files. Such data were age, sex, cause and place of injury, activity at time of injury, duration of injury before presentation to UCH, and treatment received prior to presentation. Other data obtained were initial visual acuity (IVA) and final visual acuity (FVA) at 3 months. These data had been recorded in a standardized format as practiced in the hospital for all patients.

Other clinical data obtained were level of hyphema, based on the findings at slit lamp microscopy, intraocular pressure, direct and indirect ophthalmoscopy. Level of hyphema was graded as previously described [2]. Grade 1, hyphema filling less than one third of the anterior chamber (AC); grade 2, hyphema filling one third to one half of the AC; grade 3, hyphema filling more than half of the AC, but less than the total; and grade 4, total hyphema with either red or black blood clots.

All patients were treated according to the standard eye department protocol for traumatic hyphema. The standard practice of management during this period of study was to admit all patients with traumatic hyphema, restrict activities, give cycloplegic eye drops (Guttae Atropine) and corticosteroid eye drops, with patching of the affected eye with a rigid shield. Eyes with raised intraocular pressure were treated with topical and systemic intraocular-pressure-lowering agents, commonly Guttae Timolol maleate, and oral carbonic anhydrase inhibitor. Surgical evacuation of blood (paracentesis) was performed on all eyes with black ball hyphema, corneal stain and those with intraocular pressure uncontrolled with medication [2]. Facilities for vitrectomy were not available at the time of study. A total of 336 patients (76.9%) had surgical evacuation of hyphema.

Subjects with penetrating eye injuries and non-traumatic hyphema were excluded from the study. Patients with traumatic hyphema with missing data or no follow-up and patients with follow-up of < 3 months (44 patients) were excluded from the analysis.

The follow-up data included the final corrected visual acuity (VA) and anterior and posterior segments complications. Final clinical outcome was defined as poor if visual acuity (VA) was less than 6/60. Statistical analysis was performed using the SPSS program, using simple proportions to report the findings. Approval for this study was obtained from the Joint Ethical Review Board of the University of Ibadan and University College Hospital, Ibadan.

## Results

During this study, 27% of all new patients presenting to the casualty room of this tertiary hospital with injuries of the head and neck had eye injuries alone or associated with other injuries. Of the patients with eye injuries, traumatic hyphema constituted 34.5% of all ocular and adnexial injuries that necessitating admission.

There were 516 patients admitted with a diagnosis of traumatic hyphema in a closed globe during the 10-year period. Forty-four patients were either lost to follow-up after discharge or had incomplete data, and were therefore excluded from the analysis of results. The authors reviewed 472 patients who had complete data and were followed up for a minimum of 3 months. Of the 472 patients, 338 (71.6%) were male and 134 (28.4%) were females, a ratio of 2.5:1. The demographic and ocular characteristics of the 472 patients are shown in Table 1. The mean age at presentation was 14.50 ± 7.3 year, (range 4–55 years). A majority (63.6%) of patients with traumatic hyphema were children and young adults, aged ≤ 20 years. Traumatic hyphema was more frequent in the 6–10 and 11–15 year age groups. The left eye was affected in 59.7% of cases, while the right eye was affected in 40.3% of cases. No patient presented with bilateral hyphema.

**Table 1:** Characteristics of 472 Patients with Hyphaema

Age (yrs)	n	%
0 – 5	34	7.2
6 – 10	142	30.1
11 – 15	132	28.0
16 – 20	30	6.3
21 – 25	50	10.6
26 – 30	37	7.8
31 – 40	25	5.3
41 – 50	6	1.3
> 50	16	3.4
Total	472	100
♦ Age range	4 to 55 years	
♦ Mean age	14,50 years ± 7.3 years	
♦ Sex		
Males	338	71.6
Females	134	28.4
♦ Affected Eye		
Right	190	40.3
Left	282	59.7
Full hyphaema	169/472	35.8
♦ Surgical drainage	289	61.2
♦ No with IVA worse than 6/60	298/407	73.2
♦ No with FVA worse than 6/60	143/386	37.1%

The level of hyphema varied: grade 1 (17.4%); grade 2 (20.1%); grade 3 (8.9%); and grade 4 (inclusive blackball hyphema; 53.6%). Duration of injury before presentation to the study centre ranged between 1 and 26 days with <8% of patients presenting within 24 hours of injury (Table 2).

**Table 2:** Duration of injury before presentation

Duration	No of patients	%
Less than 24 hours	37	7.8
1 – 3 days	131	27.7
4 – 7 days	117	24.8
8 – 14 days	123	26.1
> 14 days	64	13.6
Total	472	100

The place of injury is shown in Table 3. Overall, hyphema-inducing injuries that necessitated admission occurred most frequently at home (*n *= 223, 47.2%). In the 0–10 years age group, 75% (*n *= 132) of eye injuries occurred at home. Injuries occurring at school was next, occurring in 20.1% of all cases (*n *= 95). The age groups 0–10 and 11–20 years were commonly affected at school. Work-related injuries accounted for 6.5% of all cases (*n *= 36), and they occurred mostly in those aged over 20 years. The work-related injuries occurred during farming, and were caused specifically by felling of trees, harvesting of palm fruits or cocoa pods, or in welders or motor mechanics while panel-beating metals or working with nuts and bolts. A few of the injuries in the work place occurred as a result of explosions in bottling factories. Most (91%) were not wearing eye protection at the time of injury. Injuries that occurred on the roadside or street (7.6%, *n *= 36) resulted from assaults and road traffic accidents, and occurred mostly in the 21–30 years age group. Sports-related injuries that caused hyphema were infrequent, and occurred in 1.9% of all cases (*n *= 9). The sports played were football in eight cases and squash in one. The place of injury was unknown in 15 patients; 12 of these were children in the 0–10 years age group.

**Table 3:** Place of injury

	Age groups (years)				
	0 – 10	11 – 20	21 – 30	> 30	Total
	n (%)	n (%)	n (%)	n (%)	n (%)

Home	132 (75.0)	51 (31.5)	20 (23.0)	20 (42.5)	223 (47.2)
School	28 (16.9)	61 (37.6)	6 (6.9)	0 (0)	95 (20.1)
Street/Roads	0 (0)	28 (17.3)	32 (36.8)	18 (38.3)	78 (6.5)
Work	4 (2.3)	14 (8.6)	15 (17.2)	3 (6.4)	36 (7.6)
Sports/Leisure	0 (0)	2 (1.2)	2 (2.3)	5 (10.6)	9 (1.9)
Others	0 (0)	3 (1.8)	12 (13.8)	1 (2.1)	16 (3.4)
Unknown	12 (6.8)	3 (1.8)	3 (0)	0 (0)	15 (3.2)
Total	176	162	87	47	472

Agents that caused traumatic hyphema varied and were mostly sticks and belts in 45.8% of cases (*n *= 216); stones or writing materials used as missiles were other causes (15%, *n *= 71). Others were fists/elbows (9.5%, *n *= 45), iron rod, pavement/walls (8.9%, *n *= 42), fruits (cocoa pods, palm fruits) (6.8%, *n *= 32), fireworks (2.5%, *n *= 12), footballs and squash ball (3.2%, *n *= 9). Others (6.3%, *n *= 30) were swings, bolts and screws, car bumpers, gun butts and police batons.

Activities at the time of injury were play (39%, *n *= 184), corporal punishment of children by adults, with sticks or belts (21.8%, *n *= 103), assault (15.2%, *n *= 72), occupation related (10.8%, *n *= 51) and domestic (2.5%, *n *= 12), table 4. During play, falls against walls or pavements were not infrequent. Missiles of stones, writing materials and other play materials were used. Commonly, whiplash injuries were inflicted by either parents/guardians or teachers. Materials used during assault were fists, sticks, gun butts and police batons. Work tools which caused injuries were hoes, iron rods, bolts and screws.

**Table 4:** Activity at time of injury

	Age groups (years)				
	0 – 10	11 – 20	21 – 30	> 30	Total
	n (%)	n (%)	n (%)	n (%)	n (%)

Play	129 (73.3)	38 (23.5)	17 (19.5)	0 (0)	184 (39.0)
Corporal punishment	21 (11.9)	82 (50.6)	0 (0)	0 (0)	103 (21.8)
Assault/Fight	3 (1.7)	13 (8.0)	31 (35.6)	4 (8.5)	51 (10.8)
Occupational	0 (0)	16 (9.9)	32 (36.8)	24 (51.1)	72 (15.2)
Road Traffic accident	0 (0)	5 (3.1)	3 (3.4)	6 (12.8)	14 (3.0)
Domestic	1 (0.6)	2 (1.2)	2 (2.3)	7 (14.9)	12 (2.5)
Sports	0 (0)	2 (1.2)	2 (2.3)	5 (10.6)	9 (1.9)
Unknown	22 (12.5)	4 (2.4)	0 (0)	1 (2.1)	27 (5.7)
Total	176	162	87	47	472

**Figure 1:**
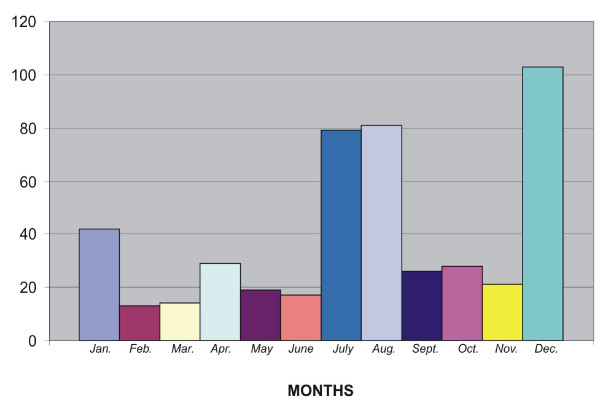
Eye injuries causing hyphaema by month.

Seasonal distribution of blunt eye injuries that resulted in hyphema is shown in Figure 1, with the maximum number of injuries occurring during the school holidays (December, August and July).

Of those whose visual acuity (VA) was recorded, initial levels were worse than 6/60 in 73.2% cases, between 6/24 and 6/60 in 16.4%, and in 10.4% of patients, the initial visual acuity (IVA) was 6/18 or better. There were 135 (35.0%) patients who had an final visual acuity (FVA) better than or equal to 6/18 at 3 months of follow-up, while it was worse than 6/60 (poor visual outcome) in 143 (37.0%) patients. Of those with poor visual outcome 105 (73.4%) had an IVA worse than 6/60. Poor visual outcome was more associated with injuries sustained in the workplace and unknown locations (Table 5).

**Table 5:** Final visual acuity at 3 months by place of injury

FVA	Place						
	Home	School	Street	Work	Sports	Others/Unknown	Total
6/4 – 6/18	61 (38.9)	37 (48.7)	10 (29.4)	17 (22.1)	7 (38.9)	3 (12.5)	135 (35.0)
6/24 – 6/60	43 (27.4)	17 (22.4)	11 (32.3)	21 (27.3)	8 (44.4)	8 (33.3)	108 (28.0)
< 6/60	53 (33.8)	22 (23.9)	13 (38.2)	39 (50.6)	3 (16.7)	13 (54.2)	143 (37.0)
Total	157	76	34	77	18	24	386

*VA not recordable in some children

Table 6 lists the ocular findings associated with traumatic hyphema 3 months after injury. These were corneal staining haziness (44.0%), lens-related factors (21.1%), post-segment lesions such as vitreous hemorrhage (17.4%), optic atrophy and retina damage, (14.7%) and phthysis bulbi (2.8%).

**Table 6:** Other ocular findings in eyes with traumatic hyphaema

	n	%
Cornea haziness	96	44.0
Lens – related problem	46	21.1
Vitreous hge	38	17.4
Optic nerve & retina	32	14.7
Phthisis bulbi	6	2.8
Total	218	100.0

## Discussion

Traumatic hyphaema was a frequent presentation and accounted for a third of those who sustained eye injuries during the period of the study. Males were more affected than females, which is similar to reports from Western Europe, America, Asia and Africa [2-9]. There was however, a relatively higher proportion of females in this series compared to other reports from Western countries [2-7]. The male preponderance reported in Europe and North America varied. The male to female ratio of patients with traumatic hyphema in 184 cases who were over 30 years of age, reported by Edwards and Layden [2], was 3.1:1. Agapitos *et al. *[3] have reported a male to female ratio of 3.6:1 among children with hyphema. Jan and Khan [9] in Pakistan reported a male to female ratio of 8:1, and among Polish children, the ratio was 4.3:1[4].

In a culture in which women traditionally live a sheltered life, the results of this study were surprising. Urbanization and cultural and socioeconomic changes that expose women to greater risks of eye injuries may explain this finding. The excess risk of severe eye injuries affecting children observed in this study had been found in several previous studies [3,5,7-9,11]; the older the children, the higher the risk of severe blunt injuries that result in hyphema [5].

Of 899 patients with an eye injury reported by Mela *et al *[11], 84% presented within 24 hours of injury or the day after, while only 7.8% patients in the present study reported for treatment within 24 hours of injury. This may be a reflection of the health service availability and/or poor patient awareness of what was available in the tertiary centre; these patients would rather have sought help elsewhere. This is corroborated by the finding that two-thirds of the patients studied admitted to having received treatment either from a chemist shop or a native doctor prior to presentation to the institution in which this study took place. The left eye was more commonly involved than the right. This may be a reflection of the fact that there are more right-handed people. Missiles or sticks used with the right hand are more likely to affect the left eye.

The home and school were the most common locations for serious eye injuries that result in hyphema, both accounting for two-thirds of the places where these injuries took place. This was the case particularly in children and adolescents. Several authors have similarly identified the home as the most common location for all types of injury [4-6], which reflects the amount of time spent in these locations, while others have reported work and sports places [11-13] to be common locations for eye injuries. The disproportionately higher figure of home- and school-related injuries in the present study may have been because of differences in population served by the hospital of study, the selective use of hospital services by children and adolescents with severe eye injuries, or a higher risk for blunt injuries for these groups in our population.

More than half of all injuries in children were as a result of intentional actions. The group chiefly at risk from thrown missiles was the 0–10 year group (30.1% of the total). Unlike in the present study, Niiraen and Ilka [14] have found that 50% of injuries were caused by other children, while a third was self-inflicted. These reported injuries took place in the absence of a caregiver. A higher number of injuries occurs during the holiday months when adult supervision is often reduced. Whereas in developed countries, toys are a common cause of injuries in the younger age groups, stones, wood, corn cobs, sticks and writing materials were often the agents of injury. Corporal punishments with sticks and belts by parents, teachers and guardians constituted over 20% of the activities that resulted in hyphema in our study. None of the children studied received their eye injuries from toys. Sticks and belts were used to beat offending children and adolescents. This is an additional risk for injuries at home and schools. Traumatic hyphema in children and adolescents secondary to corporal punishment is infrequently reported. Calzada and Kerr [15] reported seven cases, but there was no significant loss of vision, as there was in the present study.

In the adults, hyphema-inducing injuries result from different causes. In the study of McEwen [16], 69.9% of injuries were work-related, while 18.3% were sports-related. Tools and machinery either at home or the workplace were the agents of eye injuries in the study of Baker *et al. *[17]. In the present study, injuries in adults occurred in the streets, at home or at work. Activity preceeding such injuries (Table 4) were assaults or were related to work. Intentional assaults following arguments or disputes constituted a significant proportion of injuries in the group studied. A more economically friendly environment, improved employment rate and behaviour modification may reduce such injuries.

The low occurrence of sports injuries in our population suggest the low priority given to sports, and this may be due to poor information about its benefit. In recent studies from developed countries, sports related injuries have become the most common in adults [4,12,15-18], whereas work-related eye injuries used to predominate. With enforcement of appropriate legislation, work-related eye injuries have been reduced. However, increasing prosperity that makes more time available for sports and increased awareness of the health benefits of sports have increased sports injuries, up to 68% in some studies [16-18].

Work-related injuries may have been underrepresented in adults because closed eye injuries that cause hyphema were studied, whereas work-related eye injuries in a predominantly agricultural population are expected to result in penetrating eye injuries, as reported by Abraham *et al. *in Tanzania [13]. Also the rural farmer may have had limited access to the health facility where this study took place.

The visual outcome in patients who presented with traumatic hyphema was poor in 37% of cases. Only a third of patients in the present study, compared to 96% in those reported by Shiucy and Lucarelli [19] and 75% of those reported by Kearns [20], achieved visual acuity of 6/18 or better. This visual outcome is worse than in other patients in North America [15] Europe [16,17], but is similar to reports from Asia and other parts of Africa [8,9]. This may have been due to several factors. In a setting as ours, patients with hyphema presenting to hospital were likely to have severe injuries; therefore a higher proportion of those with poor visual outcome may be related to the severity of injury or the presence of other risk factors for poor outcome. Known risk factors for poor visual outcome following hyphema are delay in presentation [7-9], level of hyphema at presentation [8,11-16], associated eye damage [17,18] and abnormal hemoglobinopathy [21-24], all of which are prevalent in the study population. The latter was not routinely determined in our patients for cost reasons.

Patients with total or blackball hyphema made up about half of the group seen in the present study, similar to report Pakistan and Nigeria [8,9], but different from that reported by Edward and Layden [2]. In the latter study, only 15% of patients had grade 3 or more hyphema, while none of the 316 cases of hyphema reported by Agapitos *et al*. [3] had full hyphema. Our visual outcome was similar to that reported by Amoni and Jan [8,9], but different from outcomes in Western countries. The level of hyphema is an indicator of the severity of injury, which supports the likelihood that more severe eye injuries were presented to University College Hospital, Ibadan.

Traumatic hyphema on its own is well recognized as a serious and often vision-threatening sequela of blunt eye trauma. Hyphema may occur in isolation or more often with damage to other sites within the eye. Eyes with poor visual outcome were found to have such associated injuries in the present study. The higher rate of surgical intervention than that reported elsewhere suggests the presence of risk factors for higher intraocular pressure (IOP) and reduced egression of blood from the anterior chamber.

The findings of this study highlight that the home and school are locations in which closed globe injuries that result in hyphema occur. Such injuries still result in vision loss in about a third of cases. Unsupervised play by children and intentional actions by adults, such as corporal punishment, contributed to the increase in risk for blunt eye injuries. Differentially, females are more exposed to these injuries compared to those reported in Western countries. Prevention of blunt eye injuries requires education of children and their care givers on the potential dangers within the home and schools. Simple measures such as education, enforcement of legislation, public campaign against corporal punishment and unsupervised play could reduce these severe closed globe injuries in the communities affected. Targeted interventions to reduce stick injuries in this specific population may be needed. These prevention methods should be disseminated widely through the media, schools and all health institutions.

## Competing interests

The author declares that they have no competing interests.

## Authors' contributions

AOA conceived the idea of the study, designed the study, collected the data and drafted the manuscript.

## Pre-publication history

The pre-publication history for this paper can be accessed here:


